# Doença Arterial Coronariana Anatômica Associada à Quimioterapia em Pacientes com Câncer de Pulmão Avaliada pelo Escore Angiográfico SYNTAX

**DOI:** 10.36660/abc.20190201

**Published:** 2020-06-29

**Authors:** Qian Yang, Yundai Chen, Hai Gao, Jianzhong Zhang, Juan Zhang, Mingjie Zhang, Jing Jing, Pingjun Zhu, Hao Zhou, Shunying Hu

**Affiliations:** 1 Chinese PLA General Hospital Department of Cardiology First Medical Center Beijing China Chinese PLA General Hospital - Department of Cardiology, First Medical Center, Beijing - China; 2 Beijing An Zhen Hospital Department of Cardiology, Chaoyang-qu Beijing China Beijing An Zhen Hospital - Department of Cardiology, Chaoyang-qu, Beijing - China; 3 Unimed Scientific Inc. Wuxi China Unimed Scientific Inc., Wuxi - China; 4 Chinese PLA General Hospital Department of Oncology First Medical Center Beijing China Chinese PLA General Hospital - Department of Oncology - First Medical Center, Beijing – China

**Keywords:** Doença da Artéria Coronariana/fisiopatologia, Neoplasias Pulmonares/tratamento farmacológico, Pontuação de Propensão, Syntax Escore, Angioplastia/métodos, Fatores de Risco

## Abstract

**Fundamento:**

A doença arterial coronariana (DAC) associada à quimioterapia está se tornando um tema emergente na prática clínica. Contudo, o mecanismo subjacente da quimioterapia associada à DAC permanence incerto.

**Objetivos:**

O estudo investigou a associação entre a quimioterapia e as anomalias anatômicas ateroscleróticas das artérias coronárias dentre pacientes com cancer de pulmão.

**Métodos:**

Foram incluídos pacientes submetidos à angiografia coronária (AGC), entre 2010 e 2017, com câncer de pulmão prévio. Os fatores de risco associados à DAC e os dados sobre o câncer de pulmão foram avaliados. Avaliamos as anomalias das artérias coronárias de acordo com o escore SYNTAX (SXescore) calculado à AGC. Na análise de regressão logística, o escore SYNTAX foi classificado como alto (SXescoreALTO) se ≥22. Os dados foram analisados através de estatística descritiva e análise de regressão.

**Resultados:**

Ao todo, 94 pacientes foram incluídos no estudo. O SXescore foi mais alto no grupo com quimioterapia quando comparado com o grupo sem quimioterapia (25,25, IIQ [4,50–30,00] versus 16,50, IIQ [5,00–22,00]; p = 0,0195). A taxa do SXescoreALTO foi maior no grupo com quimioterapia do que no no grupo sem quimioterapia (58,33% versus 25,86; p = 0,0016). Tanto a análise de regressão logística univariada (OR: 4,013; 95% IC:1,655–9,731) quanto a multivariada (OR: 5,868; 95% IC:1,778–19,367) revelaram que a quimioterapia aumentou o risco de uma maior taxa do SXescoreALTO. A análise multivariada de regressão logística Stepwise mostrou que o risco para DAC anatômica mais grave aumenta com a quimioterapia como um todo em 5.323 vezes (95% IC: 2,002–14,152), e com o regime à base de platina em 5,850 vezes (95% IC: 2,027–16,879).

**Conclusões:**

A quimioterapia está associada com a complexidade e gravidade anatômica da DAC, o que pode explicar, em parte, o maior risco de DAC associada à quimioterapia dentre pacientes com câncer de pulmão. (Arq Bras Cardiol. 2020; [online].ahead print, PP.0-0)

## Introdução

As estratégias de tratameto modernas levaram à melhora nas chances de sobrevivência ao diagnóstico de câncer. Entretanto, esses ganhos têm um custo.^1^ A toxicidade cardiovascular é uma potencial complicação a curto ou longo prazo de várias terapias anticâncer e está se tornando uma das maiores preocupações no que diz respeito aos efeitos colaterais desse tipo de tratamento.^2^ As doenças do coração que podem ser induzidas por agentes quimioterápicos anticâncer incluem a disfunção cardíaca, a isquemia cardíaca, a arritmia, o AVC e a hipertensão arterial pulmonar.^1,3,4^ A doença arterial coronariana (DAC) associada à quimioterapia está se tornando um problema clínico emergente, o qual é difícil de administrar, devido as suas várias manifestações clínicas e ao complicado mecanismo fisiopatológico envolvido.5-7 Eventos coronarianos induzidos pela quimioterapia que ocorreram logo após a administração dos agentes quimioterápicos, possivelmente em decorrência de trombose aguda ou vasoespasmo, já foram relatados.^3,8^ Entretanto, a patogênese da DAC crônica associada à quimioterapia ainda não é conhecida.

O câncer de pulmão é o câncer incidental mais comum, e a principal causa de morte por câncer.^9^ A quimioterapia é um tratamento importante contra o câncer de pulmão.^10,11^Sabe-se que os agentes quimioterápicos para o câncer de pulmão, inclusive taxanos, cisplatina, carboplatina, bevacizumabe, sorafenibe e erlotinibe,^3,10,12^ causam infarto agudo do miocárdio. Desse modo, faz-se necessário investigar o efeito a longo prazo da quimioterapia sobre as alterações anatômicas da artéria coronária nos pacientes com câncer de pulmão.

A complexidade e características das lesões coronárias são preditores conhecidos de complicações periprocedimentais e de mortalidade a longo prazo.^13-15^ O escore SYNTAX (SXescore) foi desenvolvido para caracterizar prospectivamente a vasculatura coronariana por números de lesões e seus impactos funcionais, localização e complexidade.^16-18^ É uma ferramenta importante para classificar a complexidade da DAC e para a estratificação de risco de pacientes que estão sendo considerados para revascularização, e vem demonstrando valor satisfatório como um preditor de eventos cardíacos adversos significativos, incluindo a morte cardíaca. Os SXescores mais altos, indicativos de doenças mais complexas, hipoteticamente representam um desafio terapêutico maior, além de apresentarem prognósticos cardíacos potencialmente piores.^16,17,19-21^

Estudos recentes utilizaram o SXescore para quantificar a gravidade da DAC dentre pacientes com câncer, buscando examinar principalmente o efeito da radioterapia sobre a DAC.^20,21^ No presente estudo, utilizamos o SXescore para avaliar a complexidade e gravidade da DAC dentre pacientes com câncer de pulmão, a fim de investigar a relação entre a quimioterapia e a DAC. Também observamos o efeito da radioterapia e de outros fatores de risco sobre a gravidade anatômica das artérias coronárias dentre esses pacientes.

## Métodos

### Desenho e pacientes do estudo

Foi utilizado um desenho de estudo transversal hospitalar. Os pacientes do estudo foram admitidos no Hospital Geral Chinês do PLA para se submeterem à angiografia coronária (AGC) devido à suspeita de angina de peito ou estenose da artéria coronária revelada pela angiotomografia computadorizada, entre 2010 e 2017. Além disso, os pacientes deveriam ter recebido anteriormente diagnóstico definitivo de câncer de pulmão. Os pacientes que haviam sido submetidos à intervenção coronária percutânea prévia foram excluídos.

Examinamos minuciosamente os registros médicos eletrônicos dos pacientes no que diz respeito à história do câncer de pulmão, incluindo o diagnóstico, a idade à época do diagnóstico, localização e histórico do tratamento (quimioterapia e radioterapia). Revisamos o sexo, a idade à epoca da AGC, o índice de massa corporal (IMC), o histórico familiar de doenças cardiovasculares (DCVs), o uso de tabaco, a hipertensão, o diabetes, a hiperlipidemia e o perfil lipídico. Esses dados foram extraídos utilizando a plataforma de dados de pesquisa clínica criada pela Xiliu Data. Algumas informações foram checadas via telephone com os próprios pacientes ou seus familiares.

### A angiografia coronária e o SXescore

**Figura 1:**
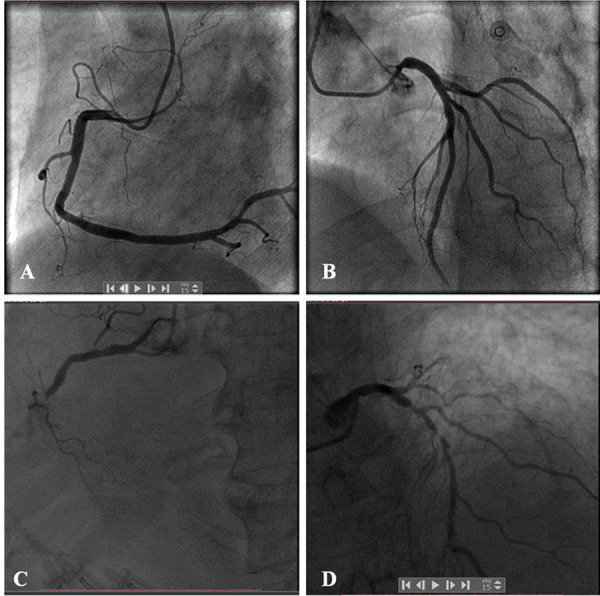
– *SXescore da artéria coronária à AGC. AGCs representativos de um paciente com SXbaixo (SXescore = 2; A–B) e um paciente com SXalto (SXescore = 38; C–D) .*

A partir do angiograma diagnóstico basal, pontuamos separadamente cada estenose coronária ≥50% em vasos com diâmetro ≥1,5 mm. Em seguida, somamos os escores para obter o SXescore global, que havíamos calculado prospectivamente utilizando o algaritmo SXescore (já totalmente descrito na literatura).^16,17,22^ Todas as variávies angiográficas pertinentes ao cálculo do SXescore foram computadas “às cegas” por 2 cardiologistas intervencionistas experientes. Quando havia divergência entre os dois cardiologistas em relação ao cálculo do SXescore de algum paciente, os mesmos discutiam o angiograma e atribuíam um SXescore comum para o paciente. Os SXescores finais foram calculados por paciente e salvos em uma base de dados dedicada. A Figura 1 mostra dois exemplos representativos de SXescores, tendo como base a AGC.

No estudo, o Sxescore de 22 foi o tercil superior. Definimos os graus do SXescore como SXbaixo (<22) ou SXalto (≥22). Por meio de análise de regressão logística, definimos que o grau do SXescore seria alto se o SXescore ≥22.

### Análise estatística

As estatísticas descritivas basais estão apresentadas como frequências e percentuais para variáveis categóricas e como média ± desvio padrão (DP) e mediana (interval interquartil [IIQ]) para variáveis contínuas. Avaliamos a normalidade dos dados utilizando o teste de assimetria e curtose. As diferenças entre grupos de estudo foram avaliadas usando o teste Qui-quadrado ou o teste exato de Fisher para dados categóricos, e o teste T foi utilizado para dados contínuos. Utilizamos o teste T para comparar as médias entre os grupos quando as variáveis estavam distribuídas normalmente, e um teste não paramétrico quando as mesmas não estavam distribuídas normalmente. O Qui-quadrado ou o teste exato de Fisher foram utilizados para examinar as diferenças para medidas categóricas. Avaliamos as relações entre a quimioterapia e a complexidade da DAC por meio de análise de regressão logística, ajustando as covariáveis relacionadas, que incluíram: idade, gênero, IMC, tabagismo, histórico familiar de DACs, hipertensão, diabetes e hiperlipidemia. As razões de probabilidade (OR) e os intervalos de confiança [ICs] em 95% foram calculados. Todos os valores de p foram bicaudais, e o nível de significância estatística de 0,05 foi adotado. Todas as análises estatísticas foram realizadas utilizando o programa SAS, versão 9.3 (SAS Institute, Inc., Cary, Carolina do Norte, EUA).

## Resultados

### Características dos pacientes

Incluímos no estudo 94 pacientes com câncer de pulmão prévio, que haviam sido submetidos à AGC no Hospital Geral Chinês do PLA, entre 2010 e 2017. Ao todo, foram incluídos 73 homens e 21 mulheres (M:F = 3,48). Dentre os participantes, oitenta e cinco foram diagnosticados com câncer de pulmão de células não pequenas, e os outros 9 pacientes com câncer de pulmão de células pequenas. Trinta e seis pacientes tinham histórico de quimioterapia. Dentre os pacientes com quimioterapia, 28 pacientes receberam regimes à base de platina. A quimioterapia à base de platina, em combinação principalmente com gencitabina ou docetaxe (e outros agentes), foi utilizada em pacientes com câncer de pulmão em células não pequenas. Em pacientes com câncer de pulmão em células pequenas, foi utilizada uma combinação quimioterápica de compostos de platina e etoposido. Um paciente recebeu antraciclina (Farmorubicina) que reconhecidamente tem um efeito cardiotóxico. Cinco pacientes receberam inibidores da tirosina quinase. Três pacientes não posuíam informações detalhadas sobre os regimes de quimioterapia. Cinquenta e oito pacientes não receberam quimioterapia.

Não foram observadas diferenças significativas em relação aos fatores de risco convencionais de DAC (hipertensão, hiperlipidemia, diabetes ou histórico de tabagismo) entre os grupos com quimioterapia e sem quimioterapia. No grupo com quimioterapia, mais pacientes receberam radioterapia do que no grupo sem quimioterapia (p < 0,0001). A variação do tempo de intervalo entre o diagnóstico de câncer e a CAG foi discrepante entre os dois grupos. As características dos pacientes estão listadas na Tabela 1.

**Tabela 1:** – Características dos pacientes estratificados por histórico de quimioterapia

Características	Grupo com quimioterapia (n = 36)	Grupo sem quimioterapia (n = 58)	Valor de p
Gênero			0,6257
Masculino	27 (75,00%)	46 (79,31%)	
Feminino	9 (25,00%)	12 (20,69%)	
Idade à AGC (anos)			0,077
<60	3 (8,33%)	13 (22,41%)	
≥60	33 (91,67%)	45 (77,59%)	
Tempo de intervalo entre o diagnóstico de câncer e a AGC (anos)			0,000
≤2	17 (47,22%)	48 (82,76%)	
>2	19 (52,78%)	10 (17,24%)	
Tipos de câncer de pulmão			0,081
Câncer de pulmão de células não pequenas	30 (83,33%)	55 (94,83%)	
Câncer de pulmão de células pequenas	6 (16,67%)	3 (5,17%)	
Regimes de quimioterapia			NA
Platina +	28 (77,78%)	--	
Inibidores de tirosina quinase apenas	5 (13,89%)	--	
Regimes não verificados	3(8,33%)	--	
Radioterapia			<0,0001
Não	19 (52,78%)	54 (93,10%)	
Sim	17 (47,22%)	4 (6,90%)	
IMC	24,81 ± 2,89	25,32 ± 2,79	0,3944
Hipertensão			0,2455
Não	12 (34,29%)	27 (46,55%)	
Sim	23 (65,71%)	31 (53,45%)	
Diabetes			0,9343
Não	28 (80,00%)	46 (80,70%)	
Sim	7 (20,00%)	11 (19,30%)	
Hiperlipidemia			0,5157
Não	29 (82,86%)	50 (87,72%)	
Sim	6 (17,14%)	7 (12,28%)	
Tabagismo			0,8938
Não	18 (51,43%)	29 (50,00%)	
Sim	17 (48,57%)	29 (50,00%)	
Consumo de álcool			0,1640
Não	45 (73,77%)	56 (62,92%)	
Sim	16 (26,23%)	33 (37,08%)	
Colesterol	4,15 ± 1,08	4,25 ± 1,10	0,6896
Trigliceríde	1,38 ± 0,71	1,49 ± 0,99	0,7905
Colesterol de lipoproteínas de baixa densidade	2,58 ± 0,96	2,48 ± 0,88	0,6999
Colesterol de lipoproteínas de alta densidade	1,17 ± 0,34	1,33 ± 0,87	0,8345

*AGC: angiografia coronária; IMC: índice de massa corporal.*

### Análise da associação entre a quimioterapia e o SXescoreALTO

Os pacientes que foram submetidos à quimioterapia desenvolveram DAC anatômica mais grave em relação àqueles que não foram submetidos à quimioterapia. O SXescore foi significativamente mais alto no grupo com quimioterapia em relação ao grupo sem quimioterapia (25,25, IIQ [4,50–30,00] versus 16,50, IIQ [5,00–22,00]; p = 0,0195). De acordo com a definicão de graus do SXescore, o percentual do SXalto foi significamente maior no grupo com quimioterapia do que no grupo sem quimioterapia (58,33% versus 25,86%; p = 0,0016). Os detalhes estão listados na Tabela 2.

**Tabela 2:** – SXescore e graus do SXescore nos pacientes com câncer de pulmão estratificados por quimioterapia ou radioterapia

Variáveis	Variável estatística	Estratificação por quimioterapia			Estratificação por radioterapia		

		Grupo com quimioterapia (n = 36)	Grupo sem quimioterapia (n = 36)	Valor de p	Grupo com radioterapia	Grupo sem radioterapia	Valor de p
SXescore							0,3045
	Média ± DP	20,00 ± 12,70	14,96 ± 10,47	0,0195	18,67 ± 12,58	16,38 ± 11,31	
	Mediana	25,25	16,50		22,00	19,00	
	Q1–Q3	4,50–30,00	5,00–22,00		5,00–30,00	5,00–25,00	
	Mín–máx	0,00–38,00	0,00–38,00		1,00–35,50	0,00–38,00	
Grau do SXescore				0,0016			0,1319
SXbaixo (<22)	N (%)	15 (41,67%)	43 (74,14%)		10 (47,62%)	48 (65,75%)	
SXalto (≥22)	N (%)	21 (58,33%)	15 (25,86%)		11 (52,38%)	25 (34,25%)	

A radioterapia é outro tratamento importante contra o câncer de pulmão. No nosso estudo, o SXescore foi mais alto no grupo com radioterapia do que no grupo sem radioterapia (22,00, IIQ [5,00–30,00] versus 19,00, IIQ [5,00–25,00]; p = 0,3045). O percentual do SXalto foi maior no grupo com radioterapia do que no grupo sem radioterapia (52,38% versus 34,25%; p = 0,1319). Entretanto, não houve diferenças significativas tanto para o SXescore quanto para a taxa do SXalto entre os grupos com radioterapia e sem radioterapia. Comparada com a radioterapia, a quimioterapia mostrou efeitos piores sobre as anomalias das artérias coronárias dentre os pacientes com câncer de pulmão. Os resultados estão apresentados na Tabela 2.

A análise de regressão logística univariada mostrou que a quimioterapia aumentou significativamente a taxa do SXalto em 4,013 vezes (95% IC: 1,655–9,731). A OR da radioterapia para o SXalto foi 2,112 (95% IC: 0,790–5,646), o que não mostrou significância estatística evidente. O tabagismo, como um fator de risco cardiovascular convencional, aumentou significativamente a taxa do SXalto em 3,182 vezes (95% IC: 1,327–7,628). A OR de outros fatores de risco cardiovasculares para o SXalto foram >1, mas não mostraram significância estatística evidente. Na análise de regressão logística multivariada, a quimioterapia aumentou o risco de DAC com anomalias anatômicas mais graves em 5,868 vezes (95% IC: 1,778-19,367). As ORs da radioterapia e do tabagismo para o SXalto foram 1,124 (95% IC: 0,286–4,416) e 3,035 (95% IC: 1,036–8,893), respectivamente. Os resultados estão apresentados na Tabela 3.

**Tabela 3:** – Análise de regressão logística pela gravidade anatômica da artéria coronária nos pacientes com câncer de pulmão

Variáveis	Modelo univariado		Modelo multivariado	

	OR (95% IC)	p	OR (95% IC)	p
Idade (anos)		0,9427		0,642
<60	Ref		Ref	
≥60	1,042 (0,343–3,161)		0,723 (0,184–2,840)	
Gênero		0,1278		0,362
Feminino	Ref		Ref	
Masculino	2,326 (0,781–7,140)		1,856 (0,490–7,021)	
IMC		0,4538		0,428
<25	Ref		Ref	
≥25	1,376 (0,597–3,168)		1,528 (0,536–4,355)	
Tabagismo		0,0095		0,043
Não	Ref		Ref	
Sim	3,182 (1,327–7,628)		3,035 (1,036–8,893)	
Histórico familiar de DAC		0,2467		0,659
Não	Ref		Ref	
Sim	2,011 (0,617–6,563)		1,379 (0,331–5,754)	
Hipertensão		0,9667		0,748
Não	Ref		Ref	
Sim	1,018 (0,437–2,372)		1,180 (0,431–3,234)	
Diabetes tipo 2		0,5338		0,501
Não	Ref		Ref	
Sim	1,393 (0,491–3,953)		1,561 (0,426–5,721)	
Hiperlipidemia		0,9732		0,616
Não	Ref		Ref	
Sim	1,021 (0,306–3,410)		0,677 (0,147–3,118)	
Intervalo de tempo entre o diagnóstico de câncer e a ACG (anos)		0,2914		0,899
≤2	Ref		Ref	
>2	1,484 (0,609–3,617)		1,075 (0,350–3,301)	
Radioterapia		0,136		0,867
Não	Ref		Ref	
Sim	2,112 (0,790–5,646)		1,124 (0,286–4,416)	
Quimioterapia		0,0021		0,004
Não	Ref		Ref	
Sim	4,013 (1,655–9,731)		5,868 (1,778–19,367)	

*IMC: índice de massa corpora; DAC: doença arterial coronariana; ACG: angiografia coronária.*

Na regressão logística multivariada Stepwise ajustada para fatores de risco relacionados à DAC (idade, gênero, IMC, tabagismo, histórico familiar de DCVs, hipertensão, diabetes e hiperlipidemia), e para fatores de risco relacionados ao câncer de pulmão (histórico de radioterapia e quimioterapia), a quimioterapia como um todo e o tabagismo aumentaram significativamente a taxa do SXalto em 5.323 vezes (95% IC: 2,002–14,152) e 3.646 vezes (95% IC:1,374–9,678), respectivamente. Além disso, detectamos que, no que diz respeito aos efeitos do regime à base de platina sobre a DAC anatômica, os resultados foram semelhantes, sendo que a OR do regime à base de platina foi 5,850 (95% IC: 2,027–16,879) e a OR do tabagismo foi 3,670 (95% IC: 1,303–10,339). Os resultados estão apresentados na Tabela 4.

**Tabela 4:** – Modelo de regressão logística multivariada Stepwise para gravidade anatômica da artéria coronária dentre pacientes com câncer de pulmão

Variáveis	OR	95% IC	Valor de p
Total de pacientes (n=94)			
Tabagismo	3,646	1,374-9,678	0,009
Quimioterapia	5,323	2,002-14,152	0,001
**Pacientes, exceto com ITQ ou RNV (n=86)**			
Tabagismo	3,670	1,303-10,339	0,14
Quimioterapia	5,850	2,027-16,879	0,007

*ITQ: inibidores da tirosina-quinase; RNV: regimes não verificados.*

## Discussão

Até onde temos conhecimento, este estudo é o primeiro a demonstrar quantitativamente que a quimioterapia está relacionada com a complexidade anatômica e a gravidade da DAC dentre os pacientes com câncer de pulmão, usando o SXescore com base nos angiogramas coronários.

A terapia antineoplásica é frequentemente dificultada pelo desenvolvimento de complicações cardiovasculares, tais como insuficiência cardíaca, infarto do miocárdio, hipertensão, tromboembolismo, prolongação do QT e bradicardia.^23^ Até o momento, as doenças do coração induzidas por quimioterapia relatadas com mais frequência são a disfunção e a insuficiência cardíaca, conforme avaliado à ecocardiografia.^1,24,25^ Os eventos da artéria coronária relacionados à quimioterapia estão se tornando problemas clínicos importantes dentre a população com câncer submetidas à quimioterapia.^5-7^ Eventos agudos da artéria coronária que ocorreram logo após a administração de agentes quimioterápicos foram relatados.^3,8^ Haugnes et al.,^26^mostraram um risco 5,7 vezes maior de DAC, e um risco de infarto do miocárdio 3,1 vezes maior, nos regimes à base de cisplatina, quando comparados com a cirurgia isolada, em um tempo médio de observação de 19 anos.^26^ O presente estudo investigou a associação entre a quimioterapia e as anomalias anatômicas das artérias coronárias dentre pacientes com câncer de pulmão.

O câncer de pulmão é o câncer incidental mais comum e é a principal causa de morte por câncer.^9^ O estudo avaliou as anomalias anatômicas das artérias coronárias através do SXescore e investigou a relação entre a quimioterapia e a complexidade anatômica da DAC dentre pacientes com câncer de pulmão. Os resultados mostraram que tanto a taxa do SXescore quanto a do SXalto foram significativamente maiores nos pacientes que foram submetidos à quimioterapia, quando comparados com pacientes que não foram. A análise de regressão logística multivariada Stepwise mostrou que o risco de CAD anatômica mais grave aumenta devido à quimioterapia como um todo em 5,323 vezes, e com os regimes à base de platina, em 5,850 vezes. Os resultados indicam que a quimioterapia está associada com a complexidade anatômica e a gravidade da DAC, o que pode, ao menos em parte, explicar a morbidade mais elevada a longo prazo da DAC associada à quimioterapia, inclusive o infarto do miocárdio.^26^ Até onde sabemos, nenhum outro estudo semelhante de grande porte detectou quantitativamente a associação entre a quimioterapia e a gravidade e complexidade anatômica da DAC dentre pacientes com câncer de pulmão.

Embora a DAC associada à quimioterapia esteja se tornando uma questão emergente, o mecanismo subjacente da DAC associada à quimioterapia permanence desconhecido. Os eventos agudos da artéria coronária que ocorreram logo após a administração de agentes quimioterápicos foram decorrentes possivelmente de trombose aguda ou vasoespasmo.^3,8^ Nosso estudo indicou que os eventos coronários a longo prazo associados à quimiterapia podem ser decorrentes de anomalias anatômicas mais graves induzidas por agentes quimioterápicos. No presente estudo, cerca de 90% dos pacientes tinham câncer de pulmão em células não pequenas, e os outros pacientes tinham câncer de pulmão em células pequenas. A maioria dos regimes de quimioterapia dos pacientes do estudo envolvia mais de um agente quimioterápico, a maioria dos quais contêm platina. A platina foi a base da quimioterapia para a maioria dos pacientes. Cinco pacientes receberam gefitinibe. No estudo, um paciente recebeu antraciclina, que é conhecida por seu efeito cardiotóxico. É razoável determinar que as células endoteliais desempenham um papel importante durante a patogênse da DAC anatômica crônica, e a lesão endotelial induzida por agentes quimioterápicos pode ser a causa central da DAC associada à quimioterapia. Cada paciente do estudo recebeu vários agentes quimioterápicos. Desse modo, foi difícil deduzir qual deles desempenhou o papel mais importante no desenvolvimento da DAC associada à quimioterapia. Já que a platina é o agente mais utilizado, talvez ela seja um dos agentes mais importantes que precisam de estudos adicionais para ilustrar o mecanismo subjacente da DAC associada à quimioterapia.

A radioterapia desempenha um papel fundamental no tratamento do câncer de pulmão.^27^ Estudos anteriores demonstraram o efeito da radiação sobre as doenças do coração.^20,28-30^ No presente estudo, ambos o SXescore e o percentual do SXalto foram mais altos no grupo com radioterapia do que no grupo sem radioterapia, mas não houve diferenças significativas entre os dois grupos. Na análise de regressão logística, a OR da radioterapia para o SXalto foi 2,112 (95% IC, 0,790–5,646), o que indica a probabilidade de a radioterapia aumentar a complexidade das artérias corronárias. Porém, os resultados não mostraram diferenças significantes. Os dados ambíguos podem ser o resultado da menor amostra de pacientes que receberam radioterapia dentre a população estudada. Com base nos resultados já mencionados, talvez possamos afirmar que a quimioterapia pode desempenhar um papel mais importante do que se pensa atualmente no que diz respeito à DAC. Entretanto, nós não podemos afirmar que a quimioterapia é pior que a radiografia em termos de DAC, principalmente devido à pequena amostra, sem dados individuais suficientes para cada agente quimioterápico. Acreditamos que os resultados são interessantes e merecem estudos futuros.

A doença cardíaca que se manifesta após o câncer pode ser decorrente de vários mecanismos: riscos cardiovasculares compartilhados entre o câncer e a doença cardiovascular, estados inflamatórios associados com malignidades e/ou efeitos cardiotóxicos do tratamento do câncer. A idade, o gênero, o uso de tabaco, o histórico familiar de DAC, a hipertensão, o diabetes tipo 2 e a hiperlipidemia são fatores de risco bastante conhecidos de DAC.^31-35^ O tabagismo é um fator de risco bem conhecido e comum tanto de DAC quanto de câncer de pulmão. No nosso estudo, metade dos pacientes com câncer de pulmão eram tabagistas, o que é consistente com os dados nacionais, mostrando que cerca de 57% dos pacientes diagnósticados com câncer de pulmão são fumantes ou ex-fumantes.^36^ No nosso estudo, outros fatores de risco cardiovascular também aumentaram a gravidade da DAC, mas aqueles fatores de risco não demonstraram significância estatística evidente para o aumento do SXescore. Por outro lado, o tabagismo mostrou mais efeitos significativos sobre o aumento do risco para SXalto em 3.646 vezes.

Além disso, o tempo de duração do câncer de pulmão pode desempenhar um papel na progressão da DAC. No estudo, coletamos dados do tempo de intervalo entre o diagnóstico do câncer e a AGC. Embora o tempo de intervalo entre o diagnóstico do câncer e a AGC tenha sido discrepante entre os dois grupos (possivelmente por se tratar de um estudo retrospectivo de pequeno porte), o modelo logístico multivariado com ajuste adicional para a variável tempo de intervalo ainda mostrou diferenças significativas na gravidade da DAC entre os pacientes com quimioterapia e aqueles sem quimioterapia.

Nosso estudo tem várias limitações. Em primeiro lugar, trata-se de um estudo unicêntrico com uma amostra pequena, realizado com uma população específica de pacientes que haviam tido câncer de pulmão e necessitado de AGC devido à suspeita de DAC grave. Um número menor de pacientes recebeu radioterapia. Dentre os 94 pacientes do estudo, 21 pacientes costumavam receber radioterapia. Em particular, apenas 4 pacientes (6,9%) tinham histórico de radioterapia no grupo sem quimioterapia. Os resultados da amostra da população de pacientes, por ser pequena e específica, podem ser desviantes. Em segundo lugar, este foi um estudo retrospectivo, que pode carecer de algumas informações valiosas acerca dos pacientes. Seria útil saber o estágio do câncer de pulmão na apresentação inicial. Aqueles que receberam quimioterapia podem ter tido uma doença mais avançada e, consequentemente, mais inflamação, por um período mais longo de tempo, o que pode causar aterosclerose e contribuir para os resultados observados neste estudo. Entretanto, nós não recebemos dados tão amplos sobre os pacientes. Em terceiro lugar, nós não investigamos se SXescore estava associado com eventos cardiovasculares a longo prazo nos pacientes do estudo. Estudos clínicos adicionais prospectivos e em larga escala podem ser necessários para verificar o efeito da quimioterapia sobre a anomalia anatômica da DAC e o mecanismo subjacente da DAC associada à quimioterapia.

## Conclusões

Em resumo, o presente estudo demonstra que a quimioterapia está associada à gravidade e complexidade anatômica da DAC a longo prazo. Os resultados podem explicar em parte o fato de pacientes com câncer e histórico de quimioterapia apresentarem um risco mais elevado de eventos da artéria coronária, quando comparados com pacientes sem histórico de quimioterapia. Entretanto, devido às limitações, um estudo prospectivo de larga escala, bem como pesquisas futuras fisiopatológicas e moleculares, serão necessários para melhor ilustrar a associação entre a quimioterapia e a DAC, e o mecanismo subjacente da CAD associada à quimioterapia.
