# Supplementary material for: Doença Arterial Coronariana Anatômica Associada à Quimioterapia em Pacientes com Câncer de Pulmão Avaliada pelo Escore Angiográfico SYNTAX
Source: Arq Bras Cardiol. 2020 Jun 29;114(6):1004–12. [Article in Portuguese] doi: 10.36660/abc.20190201 (PMC8416133; doi:10.36660/abc.20190201)
Supplement: Translation

# Translation: Chemotherapy-Related Anatomical Coronary-Artery Disease in Lung Cancer Patients Evaluated by Coronary-Angiography SYNTAX Score

**Authors:** Qian Yang, Yundai Chen, Hai Gao, Jianzhong Zhang, Juan Zhang, Mingjie Zhang, Jing Jing, Pingjun Zhu, Hao Zhou, Shunying Hu

**Affiliations:** 1 China Chinese PLA General Hospital - Department of Cardiology, First Medical Center, Beijing - China; 2 China Beijing An Zhen Hospital - Department of Cardiology, Chaoyang-qu, Beijing - China; 3 China Unimed Scientific Inc., Wuxi - China; 4 China Chinese PLA General Hospital - Department of Oncology - First Medical Center, Beijing – China

**Keywords:** Coronary Artery Disease/physiopathology, Lung Neoplasms/drug therapy, Lung Neoplasms/complications, Propensity Score, Score Syntax, Angioplasty/methods, Risk Factors

## Abstract

**Background:**

Chemotherapy-related coronary artery disease (CAD) is becoming an emerging issue in clinic. However, the underlying mechanism of chemotherapy-related CAD remains unclear.

**Objective:**

The study investigated the association between chemotherapy and atherosclerotic anatomical abnormalities of coronary arteries among lung cancer patients.

**Methods:**

Patients undergoing coronary angiography (CAG) between 2010 and 2017, who previously had lung cancer, were examined. Risk factors associated with CAD and information about lung cancer were evaluated. We assessed coronary-artery abnormalities by SYNTAX score (SXscore) based on CAG. In logistic-regression analysis, we defined high SXscore (SXhigh) grade as positive if ≥22. Data were analyzed through descriptive statistics and regression analysis.

**Results:**

A total of 94 patients were included in the study. The SXscore was higher in the chemotherapy group than in the non-chemotherapy group (25.25, IQR [4.50–30.00] vs. 16.50, IQR [ 5.00–22.00], p = 0.0195). The SXhigh rate was greater in the chemotherapy group than in the non-chemotherapy group (58.33% vs. 25.86; p = 0.0016). Both univariate (OR:4.013; 95% CI:1.655–9.731) and multivariate (OR:5.868; 95% CI:1.778–19.367) logistic-regression analysis revealed that chemotherapy increased the risk of greater SXhigh rates. Multivariate stepwise logistic-regression analysis showed the risk of more severe anatomical CAD is increased by chemotherapy as a whole by 5.323 times (95% CI: 2.002–14.152), and by platinum-based regimens by 5.850 times (95% CI: 2.027–16.879).

**Conclusions:**

Chemotherapy is associated with anatomical complexity and severity of CAD, which might partly account for the higher risk of chemotherapy-related CAD among lung cancer patients. (Arq Bras Cardiol. 2020; [online].ahead print, PP.0-0)

## Introduction

Modern treatment strategies have led to an improvement in the chances of surviving a diagnosis of cancer. However, these gains can come at a cost.^1^ Cardiovascular toxicity is a potential short- or long-term complication of various anticancer therapies and is becoming one of the most concerning side effects of anti-cancer therapy.^2^ Heart conditions that may be induced by anticancer chemotherapeutic agents include cardiac dysfunction, cardiac ischemia, arrhythmia, stroke and pulmonary-artery hypertension.^1,3,4^Chemotherapy-related coronary artery disease (CAD) is becoming an emerging clinical problem difficult to manage due to various clinical manifestation and complicated pathophysiological mechanisms.^5-7^ Chemotherapy-induced coronary-artery events that occurred shortly after administration of chemotherapeutic agents, possibly due to acute thrombosis or vasospasm, have been reported.^3,8^ However, the pathogenesis of chronic chemotherapy-related CAD remains unclear.

Lung cancer is the most common incident cancer and the leading cause of cancer death.^9^ Chemotherapy is an important treatment for this disease.^10,11^ Chemotherapeutic agents for lung cancer, including taxanes, cisplatin, carboplatin, bevacizumab, sorafenib and erlotinib^3,10,12^ are known to cause acute myocardial infarction (AMI). It is important to investigate the long term effect of chemotherapy on anatomical changes of coronary artery among lung cancer patients.

Complexity and lesion characteristics of the coronary artery are well-recognized predictors of periprocedural complications and long-term mortality.^13-15^ The SYNTAX score (SXscore) was developed to prospectively characterize the coronary vasculature by number of lesions and their functional impacts, locations and complexity.^16-18^ It is an important tool for grading complexity of coronary artery disease (CAD) and for risk-stratifying patients who are being considered for revascularization. In addition, it has demonstrated good value as a predictor of major adverse cardiac events, including cardiac death. Higher SXscores, indicative of more-complex diseases, are hypothesized to represent a greater therapeutic challenge and to pose potentially worse cardiac prognoses.^16,17,19-21^

Recent studies used SXscore to quantify the severity of CAD among cancer patients, which looked mostly at the effect of radiotherapy on CAD.^20,21^ In the present study, we used SXscore to evaluate the complexity and severity of CAD among lung cancer patients to investigate the relationship between chemotherapy and CAD. We also observed the effect of radiotherapy and other risk factors on anatomical severity of coronary arteries among those patients.

## Methods

### Study design and patients

We used a hospital-based cross-sectional study design. The study patients were admitted to Chinese PLA General Hospital to undergo coronary angiography (CAG) due to suspected angina pectoris or stenosed coronary artery, showed by computer tomography angiography, between 2010 and 2017. Furthermore, the patients should have previously received definite diagnoses of lung cancer. Patients who had previously undergone percutaneous coronary intervention were excluded.

We thoroughly examined the patients’ electronic medical records for history of lung cancer, including diagnosis, age at time of diagnosis, location and treatment history (chemotherapy and radiotherapy). We reviewed sex, age at time of CAG, body mass index (BMI), family history of cardiovascular diseases (CVDs), tobacco use, hypertension, diabetes, hyperlipidemia and lipid profile. These data were extracted using a clinical-research data platform created by Xiliu Data. Some data were checked by telephone with the patients themselves or their families.

### Coronary angiography and SXscore

From the baseline diagnostic angiogram, we separately scored each coronary lesion with stenosis ≥50% in a vessel ≥1.5 mm diameter. Next, we added the scores to provide the overall SXscore, which we had calculated prospectively using the SXscore algorithm (described in full elsewhere in the literature).^16,17,22^ All angiographic variables pertinent to SXscore calculation were computed by two blinded experienced interventional cardiologists. When the SXscore of each patient differed between the two cardiologists, they would discuss the angiogram and come up with a common SXscore for each patient. Final SXscores were calculated per patient and saved in a dedicated database. Two representative examples with SXscores based on CAG are shown in Figure 1.

**Figure 1:** – *SXscore of coronary artery based on CAG. Representative CAGs of a patient with SXlow (SXscore = 2; A–B) and a patient with SXhigh (SXscore = 38; C–D).*

In the study, a Sxscore of 22 was the upper tertile. We defined SXscore grades as SXlow (<22) or SXhigh (≥22). Through logistic-regression analysis, high SXscore grade was determined as positive if SXscore ≥22.

### Statistical analysis

Baseline descriptive statistics are presented as frequencies and percentages for categorical variables and mean ± standard deviation (SD) and median (interquartile range [IQR]) for continuous variables. The normality of data was assessed using the Skewness and Kurtosis normality test. Differences between the study groups were assessed by chi-square test or Fisher’s exact test for categorical data, and by student’s t test for continuous data. We used student’s t test to compare the groups’ means when variables were normally distributed, and a non-parametric test when they were not normally distributed. Chi-square or Fisher’s exact test were used to examine differences for categorical measures. We assessed the relationships between chemotherapy and CAD complexity by logistic-regression analysis, adjusting related covariates that included age, gender, BMI, smoking, family history of CVDs, hypertension, diabetes and hyperlipidemia. Odds ratios (ORs) and 95% confidence intervals [CIs] were calculated. P values were 2-tailed, and we set the level of significance at 0.05. All statistical analyses were performed using SAS software, version 9.3 (SAS Institute, Inc., Cary, North Carolina, USA).

## Results

### Patient characteristics

A total of 94 patients who had previously had lung cancer and who underwent CAG at Chinese PLA General Hospital, between 2010 and 2017, were included in the study. Out of these, 73 were males and 21 females (M:F = 3.48). Eighty-five patients were diagnosed with non-small cell lung cancer, and the other 9 patients with small cell lung cancer. Thirty-six patients had histories of chemotherapy. Among the patients with chemotherapy, 28 patients received platinum-based regimens. Platinum-based regimens combining gemcitabine or docetaxel, and other agents, were used in non-small cell lung cancer patients, and double-platinum chemotherapy combined with etoposide was used in small cell lung cancer patients. One patient received anthracycline (pharmorubicin), which is known to have cardiac toxicity. Five patients received tyrosine kinase inhibitors (gefitinib). Three patients lacked detailed information about chemotherapy regimens. Fifty-eight patients did not receive any chemotherapy.

There were no significant differences regarding conventional CAD risk factors (hypertension, hyperlipidemia, diabetes or smoking history) between the chemotherapy and non-chemotherapy groups. In the chemotherapy group, more patients took radiotherapy than in the non-chemotherapy group (p < 0.0001). The time interval range from cancer diagnosis to CAG was discrepant between the two groups. Patient characteristics are listed in Table 1.

**Table 1:** – Patient characteristics stratified by history of chemotherapy

Characteristic	Chemotherapy group (n = 36)	Non-chemotherapy group (n = 58)	p value
Gender			0.6257
Male	27 (75.00%)	46 (79.31%)	
Female	9 (25.00%)	12 (20.69%)	
Age at CAG (years)			0,077
<60	3 (8,33%)	13 (22,41%)	
≥60	33 (91.67%)	45 (77,59%)	
Interval time from cancer diagnosis to CAG (years)			0.000
≤2	17 (47.22%)	48 (82.76%)	
>2	19 (52.78%)	10 (17.24%)	
Types of lung cancer			0.081
Non-small cell lung cancer	30 (83.33%)	55 (94.83%)	
Small cell lung cancer	6 (16.67%)	3 (5.17%)	
Regimens of chemotherapy			NA
Platinum +	28 (77.78%)	--	
Tyrosine kinase inhibitors only	5 (13.89%)	--	
Not-verified regimens	3(8.33%)	--	
Radiotherapy			<0.0001
No	19 (52.78%)	54 (93.10%)	
Yes	17 (47.22%)	4 (6.90%)	
BMI	24.81 ± 2.89	25.32 ± 2.79	0.3944
Hypertension			
No	12 (34.29%)	27 (46.55%)	
Yes	23 (65.71%)	31 (53.45%)	
Diabetes			0.9343
No	28 (80.00%)	46 (80.70%)	
Yes	7 (20.00%)	11 (19.30%)	
Hyperlipidemia			0.5157
No	29 (82.86%)	50 (87.72%)	
Yes	6 (17.14%)	7 (12.28%)	
Smoking			0.8938
No	18 (51.43%)	29 (50.00%)	
Yes	17 (48.57%)	29 (50.00%)	
Alcohol consumption			0.1640
No	45 (73.77%)	56 (62.92%)	
Yes	16 (26.23%)	33 (37.08%)	
Cholesterol	4.15 ± 1.08	4.25 ± 1.10	0.6896
Triglyceride	1.38 ± 0.71	1.49 ± 0.99	0.7905
Low-density lipoprotein cholesterol	2.58 ± 0.96	2.48 ± 0.88	0.6999
High-density lipoprotein cholesterol	1.17 ± 0.34	1.33 ± 0.87	0.8345

*BMI: body mass index; CAG: coronary angiography.*

### Analysis of association between chemotherapy and high SXscore

Patients who underwent chemotherapy developed more-severe anatomical CAD than those who did not undergo chemotherapy. The SXscore was significantly higher in the chemotherapy group than in the non-chemotherapy group (25.25,IQR [4.50–30.00] vs. 16.50, IQR[5.00–22.00]; p = 0.0195). According to the SXscore grade definition, the percentage of SXhigh was significantly higher in the chemotherapy group than in the non-chemotherapy group (58.33% vs. 25.86%; p = 0.0016). Details are shown in Table 2.

**Table 2:** – SXscore and SXscore grades in lung cancer patients stratified by chemotherapy or radiotherapy

Variables	Statistical variable	Chemotherapy stratification			Radiotherapy stratification		

		Chemotherapy group	Non-chemotherapy group (n = 58)	p value	Radiotherapy group (n = 21)	Non-radiotherapy group (n = 73)	p value
SXscore							0.3045
	Mean ± SD	20.00 ± 12.70	14.96 ± 10.47	0.0195	18.67 ± 12.58	16.38 ± 11.31	
	Median	25.25	16.50		22.00	19.00	
	Q1–Q3	4.50–30.00	5.00–22.00		5.00–30.00	5.00–25.00	
	Min–max	0.00–38.00	0.00–38.00		1.00–35.50	0.00–38.00	
SXscore grade				0.0016			0.1319
SXlow (<22)	N (%)	15 (41.67%)	43 (74.14%)		10 (47.62%)	48 (65.75%)	
SXhigh (≥22)	N (%)	21 (58.33%)	15 (25.86%)		11 (52.38%)	25 (34.25%)	

Radiotherapy is another important treatment for lung cancer. In our study, the SXscore was higher in the radiotherapy group than in the non-radiotherapy group (22.00, IQR[ 5.00–30.00] vs. 19.00, IQR[5.00–25.00]; p = 0.3045). The percentage of SXhigh was higher in the radiotherapy group than in the non-radiotherapy group (52.38% vs. 34.25%; p = 0.1319). However, there was no significant difference for either SXscore or SXhigh rates between the radiotherapy and non-radiotherapy groups. Compared with radiotherapy, chemotherapy showed worse effects on anatomical abnormalities of coronary arteries among lung cancer patients. Results are presented in Table 2.

Univariate logistic-regression analysis showed that chemotherapy significantly increased the SXhigh rate by 4.013 times (95% CI:1.655–9.731). The OR of radiotherapy for SXhigh was 2.112 (95% CI: 0.790–5.646), which showed no obvious statistical significance. Smoking as a conventional cardiovascular risk factor was shown to significantly increase the SXhigh rate by 3.182 times (95% CI:1.327–7.628). The ORs of other cardiovascular risk factors for SXhigh were >1, but showed no obvious statistical significance. In multivariate logistic-regression analysis, chemotherapy was shown to increase the risk of CAD with more-severe anatomical abnormalities by 5.868 times (95% CI: 1.778-19.367). The ORs of radiotherapy and smoking for SXhigh were 1.124 (95% CI: 0.286–4.416) and 3.035 (95% CI: 1.036–8.893), respectively. Results are shown in Table 3.

**Table 3:** – Logistic-regression analysis for anatomical severity of the coronary artery in lung cancer patients

Variable	Univariate model		Multivariate model	

	OR (95% CI)	p	OR (95% CI)	p
Age (years)		0.9427		0.642
<60	Ref		Ref	
≥60	1.042 (0.343–3.161)		0.723 (0.184–2.840)	
Gender		0.1278		0.362
Female	Ref		Ref	
Male	2.326 (0.781–7.140)		1.856 (0.490–7.021)	
BMI		0.4538		0.428
<25	Ref		Ref	
≥25	1.376 (0.597–3.168)		1.528 (0.536–4.355)	
Smoking		0.0095		0.043
No	Ref		Ref	
Yes	3.182 (1.327–7.628)		3.035 (1.036–8.893)	
Family history of CAD		0.2467		0.659
No	Ref		Ref	
Yes	2.011 (0.617–6.563)		1.379 (0.331–5.754)	
Hypertension		0.9667		0.748
No	Ref		Ref	
Yes	1.018 (0.437–2.372)		1.180 (0.431–3.234)	
Type II diabetes		0.5338		0.501
No	Ref		Ref	
Yes	1.393 (0.491–3.953)		1.561 (0.426–5.721)	
Hyperlipidemia		0.9732		0.616
No	Ref		Ref	
Yes	1.021 (0.306–3.410)		0.677 (0.147–3.118)	
Interval time from cancer diagnosis to CAG (years)		0.2914		0.899
≤2	Ref		Ref	
>2	1.484 (0.609–3.617)		1.075 (0.350–3.301)	
Radiotherapy		0.136		0.867
No	Ref		Ref	
Yes	2.112 (0.790–5.646)		1.124 (0.286–4.416)	
Chemotherapy		0.0021		0.004
No	Ref		Ref	
Yes	4.013 (1.655–9.731)		5.868 (1.778–19.367)	

*BMI: body mass index; CAD: coronary artery disease; CAG: coronary angiography.*

In multivariate stepwise logistic regression adjusted for related CAD risk factors (age, gender, BMI, smoking, family history of CVDs, hypertension, diabetes and hyperlipidemia) and lung cancer–related risk factors (history of radiotherapy and chemotherapy), chemotherapy as a whole and smoking were shown to significantly increase the SXhigh rate by 5.323 times (95% CI: 2.002–14.152) and by 3.646 times (95% CI: 1.374–9.678), respectively. Moreover, we detected that the effects of platinum-based regimen on anatomical CAD were similar: the OR of platinum-based regimen was 5.850 (95% CI: 2.027–16.879), and the OR of smoking was 3.670 (95% CI: 1.303–10339). Results are shown in Table 4.

**Table 4:** – Multivariate stepwise logistic-regression model for anatomical severity of the coronary artery among lung cancer patients

Variables	OR	95% CI	p value
Total patients (n=94)			
Smoking	3.646	1.374-9.678	0.009
Chemotherapy	5.323	2.002-14.152	0.001
Patients except with TKI or NVR (n=86)			
Smoking	3.670	1.303-10.339	0.14
Chemotherapy	5.850	2.027-16.879	0.007

*TKI: Tyrosine kinase inhibitors; NVR: Not verified regimens*

## Discussion

To the best of our knowledge, this study is the first to quantitatively demonstrate that chemotherapy is related to anatomical complexity and severity of CAD among lung cancer patients, using SXscore based on coronary angiograms.

Antineoplastic therapy is frequently hindered by the development of cardiovascular complications such as heart failure, myocardial infarction, hypertension, thromboembolism, QT prolongation and bradycardia.^23^ Until now, the most often reported chemotherapy-induced heart conditions have been cardiac dysfunction and heart failure, as evaluated by echocardiography.^1,24,25^ Chemotherapy-related coronary-artery events are becoming important clinical problems among the cancer population who received chemotherapy.^5-7^ Acute coronary-artery events that occurred shortly after administration of chemotherapeutic agents were reported.^3,8^ Haugnes et al.^26^ showed a 5.7-fold higher risk of CAD and a 3.1-fold higher risk of myocardial infarction with cisplatin-based regimens compared with surgery alone, in a median observation time of 19 years.^26^ The present study investigated the association between chemotherapy and anatomical abnormalities of coronary arteries among lung cancer patients.

Lung cancer is the most common incident cancer and the leading cause of cancer death.^9^ The study assessed anatomical abnormalities of coronary arteries by the SXscore and investigated the relationship between chemotherapy and anatomical complexity of CAD among lung cancer patients. Results showed that both SXscore and SXhigh rates were significantly greater in patients who underwent chemotherapy compared with patients who did not. Multivariate stepwise logistic-regression analysis showed that the risk of more severe anatomical CAD is increased by chemotherapy as a whole by 5.323 times, and by platinum-based regimens by 5.850 times. The results indicate that chemotherapy is associated with the anatomical complexity and severity of CAD, which may at least partly explain the long-term higher morbidity of chemotherapy-related CAD, including myocardial infarction.^26^ To our knowledge, no similar large study has quantitatively detected the association between chemotherapy and anatomical complexity and severity of CAD among lung cancer patients.

Although chemotherapy-related CAD is becoming an emerging issue, the underlying mechanism of chemotherapy-related CAD remains unclea. Acute coronary-artery events that occurred shortly after administration of chemotherapeutic agents were possibly due to acute thrombosis or vasospasm.^3,8^Our study indicated that long-term chemotherapy-related coronary events may be due to more severe anatomical abnormalities induced by chemotherapeutic agents. In the present study, about 90% of the study patients are non-small cell lung cancer patients, and the others are small cell lung cancer patients. Most of chemotherapy regimens for the study patients involved more than one chemotherapeutic agent, most of which contained platinum. In fact, platinum was the base of chemotherapy for most of the patients. In the study, five patients received gefitinib and one patient received anthracycline, which is known to have cardiac toxicity. It is reasonable to determine that endothelial cells play an important role during the pathogenesis of chronic anatomical CAD. Besides, chemotherapeutic agents-induced endothelial injuries might be the core cause of chemotherapy-related CAD. Each study patient took various chemotherapeutic agents. Thus, it was difficult to infer which played the most important role in the progress of chemotherapy-related CAD. Since platinum is the most used agent, it may be one of the most important agents to be further studied for illustrating the underlying mechanisms of chemotherapy-related CAD.

Radiotherapy plays a major role in the management of lung cancer.^27^ Previous studies have shown the effect of radiation on heart diseases.^20,28-30^ In the present study, both the SXscore and the SXhigh percentage were greater in the radiotherapy group in relation to the non-radiotherapy group. Nevertheless, no significant differences were observed between the two groups. In logistic-regression analysis, the OR of radiotherapy for the SXhigh was 2.112 (95% CI: 0.790–5.646), which means that radiotherapy is likely to increase the anatomical complexity of coronary arteries. However, the results could not show significant differences. The ambiguous results may be due to the smaller sample of patients receiving radiotherapy in the study population. Based on the results mentioned, we could say that chemotherapy may play a more important role than is currently thought in terms of CAD. However, it was not possible to determine that chemotherapy is worse than radiotherapy in terms of CAD, particularly due to the small sample and the lack of enough individual data for each chemotherapeutic agent. Still, we believe the results are interesting and deserve further study.

Heart disease manifesting after cancer may be due to several mechanisms: shared cardiovascular risks between cancer and cardiovascular disease, inflammatory states associated with malignancies and/or cardiotoxic effects of cancer therapy. Age, gender, tobacco use, family history of CAD, hypertension, type II diabetes and hyperlipidemia are the well-known risk factors for CAD.^31-35^ Smoking is a well-known common risk factor for both CAD and lung cancer. In our study, half of lung cancer patients were smokers, which is consistent with national data, showing that about 57% of lung cancer diagnosed patients were either current or former smokers.^36^ In the study, other cardiovascular risk factors have shown to be likely to increase the severity of CAD. However, those risk factors did not show obvious statistical significance for increasing the SXscore. On the other hand, smoking showed a more significant effect, by increasing the risk for SXhigh by 3.646 times.

Moreover, the length of time of lung cancer may play a role in the progression of CAD. In the study, we collected the data on the time interval between cancer diagnosis and CAG. Although the time interval between cancer diagnosis and CAG was discrepant between the two groups (possibly because this is a small retrospective study), the multivariate logistic analysis, adjusted for the time interval variable, showed a significant difference regarding CAD severity between patients with chemotherapy and those without chemotherapy.

Our study has several limitations. First, it was a small sample single-center study, performed among a specific population of patients, who had had lung cancers and who required CAG due to suspected severe CAD. A lower number of patients received radiotherapy: among the 94 study patients, 21 used to receive radiotherapy. In particular, only 4 patients (6.9%) had a history of radiotherapy in the non-chemotherapy group. Therefore, the results from this specific small sample may be deviant. Second, it was a retrospective study, thus some valuable information on the study patients might be lacking. For instance, it would be helpful to know the stage of lung cancer at initial presentation, since those who received chemotherapy could have had more advanced disease and, consequently, more inflammation for a longer period of time, which may promote atherosclerosis and contribute to the results observed. However, we were not able to obtain such comprehensive information on the patients. Third, we did not investigate whether the SXscore was associated with long-term cardiovascular events in the study patients. Additional prospective, large-scale clinical studies may be required to verify the effect of chemotherapy on the anatomical abnormality of CAD and the underlying mechanisms of chemotherapy-related CAD.

## Conclusions

In brief, the present study demonstrates that chemotherapy is associated with long-term anatomical complexity and CAD severity. The results could partly explain why cancer patients with a history of chemotherapy are at higher risk of suffering coronary events compared to those with no history of chemotherapy. However, due to the limitations mentioned, a large-scale prospective study, as well as further pathophysiological and molecular researches, are needed to further illustrate the association between chemotherapy and CAD, and the underlying mechanisms of chemotherapy-related CAD.
